# Study of Cardiovascular Dysfunction in Cirrhosis and Its Correlation With Severity at a Tertiary Care Hospital

**DOI:** 10.7759/cureus.109584

**Published:** 2026-05-25

**Authors:** Bijaya K Behera, Romanchit Upadhyaya, Manasi Das, Sanatan Behera

**Affiliations:** 1 Medicine, Srirama Chandra Bhanja (SCB) Medical College and Hospital, Cuttack, IND; 2 Internal Medicine, Srirama Chandra Bhanja (SCB) Medical College and Hospital, Cuttack, IND; 3 Department of Neurology, IMS and SUM Hospital, Campus II, Cuttack, IND; 4 Gastroenterology, Srirama Chandra Bhanja (SCB) Medical College and Hospital, Cuttack, IND

**Keywords:** cardiovascular dysfunction, child-turcotte-pugh score, cirrhosis, cirrhotic cardiomyopathy, diastolic dysfunction, echocardiography

## Abstract

Background: Cirrhosis of the liver is accompanied by systemic circulatory changes that can give rise to cardiovascular changes collectively termed cirrhotic cardiomyopathy. While these changes can be clinically silent, they can aggravate conditions, especially in the later stages of liver disease. The current study aims to examine cardiovascular dysfunction in patients with cirrhosis and its relationship with disease severity.

Methods: Between April 2023 and June 2024, a study was carried out at Srirama Chandra Bhanja Medical College and Hospital in Cuttack, involving 103 participants. Each patient underwent a series of evaluations, including echocardiography, laboratory tests, electrocardiography (ECG), and clinical examinations. Additionally, the Child-Turcotte-Pugh (CTP) classification was utilized to assess the severity of cirrhosis. To evaluate cardiac dysfunction, cardiology blood tests, ECG, and echocardiography were performed to measure the left ventricular ejection fraction.

Results: The mean age of the study population was 44.22 ± 10.97 years (n = 103), with 66 male (64%) and 37 female (36%) participants. Alcohol-related liver disease was the most common etiology (72 patients, 69.9%). Diastolic dysfunction was the most frequent cardiac abnormality and increased significantly with worsening CTP class (p < 0.05). Systolic dysfunction was less common, with most patients demonstrating preserved ejection fraction. ECG abnormalities, including QT interval prolongation, were observed in a subset of patients. Given the predominance of alcohol-related cirrhosis, the potential independent contribution of alcohol to cardiac abnormalities should also be considered.

Conclusion: Cardiovascular dysfunction, particularly diastolic dysfunction, is common in cirrhotic patients and correlates with the severity of liver disease. However, the high proportion of alcohol-related cirrhosis in the study population may have influenced the observed cardiac findings. Routine cardiovascular assessment may facilitate early detection and improved clinical management.

## Introduction

The definitive features of cirrhosis, the most advanced stage of chronic liver disease, include diffuse hepatic fibrosis, regenerative nodules, and changes in the architecture of the liver. It comes with a variety of systemic complications affecting the kidneys, lungs, brain, and heart and blood vessels. Of these, in the past few years, research interest has grown for the cardiovascular complications due to the high level of morbidity and mortality in patients with advanced liver disease [1]. Patients with cirrhosis have been described to have a hyperdynamic circulatory state, but many of the cardiac problems can remain quiet until they are brought to the forefront due to some physiological demand, such as infection, bleeding, transjugular intrahepatic portosystemic shunt, or liver transplantation [2].

Cirrhotic cardiomyopathy is a syndrome described in patients with cirrhosis characterized by impaired cardiac response to stress, diastolic dysfunction, and electrophysiological abnormalities in the absence of known primary cardiac disease. Although diagnostic criteria have evolved over time and remain variable across studies, cirrhotic cardiomyopathy is increasingly recognized as a significant cardiovascular complication of advanced liver disease. In cirrhosis, peripheral vasodilation mediated by nitric oxide and other vasoactive substances leads to activation of compensatory neurohormonal systems, including the sympathetic nervous system, vasopressin pathway, and renin-angiotensin-aldosterone system. These changes contribute to hyperdynamic circulation, sodium and water retention, and cardiovascular dysfunction [3,4].

Diastolic dysfunction is one of the earliest and most frequently reported manifestations of cirrhotic cardiomyopathy. Structural and functional myocardial changes, such as myocardial hypertrophy, interstitial fibrosis, and impaired ventricular relaxation, contribute to abnormal diastolic filling patterns detectable on echocardiography [5]. Furthermore, patients with cirrhosis have reported electrophysiological abnormalities, such as prolonged QT interval and chronotropic incompetence, which emphasize the intricate cardiovascular complications associated with this condition [6].

The extent of cardiovascular dysfunction has been shown to correlate with the extent of liver disease. The clinical scoring systems, Child-Pugh and model for end-stage liver disease, assist in determining the prognosis and severity of cirrhosis. Studies have shown that patients with advanced cirrhosis, particularly those with Child-Pugh B and C, are at a greater risk of developing significant cardiac issues compared with patients with early-stage disease [7]. Because the detection of cardiovascular dysfunction may alter treatment options and affect prognosis and perioperative liver transplant risk, it is especially relevant in this group of patients [8].

The present study was conducted to evaluate cardiovascular dysfunction in patients with liver cirrhosis and to assess its association with the severity of liver disease based on the Child-Turcotte-Pugh (CTP) classification. The primary objective was to evaluate the prevalence of diastolic dysfunction across different stages of cirrhosis, while secondary objectives included assessment of systolic dysfunction, corrected QT (QTc) prolongation, and pulmonary hypertension using electrocardiographic and echocardiographic parameters.

## Materials and methods

Study design

This observational cross-sectional study was conducted at the Srirama Chandra Bhanja (SCB) Medical College and Hospital in Cuttack, in the departments of cardiology, hepatology, and general medicine. The 15-month investigation was conducted between April 2023 and June 2024.

Study population and sample size

The sample size was calculated using the standard formula:

$n {=} \frac{4pq}{d^2}$

where p represents the estimated prevalence, q = 1 - p, and d denotes the acceptable margin of error.

The prevalence of cardiovascular abnormalities among patients with chronic liver disease was assumed to be 40% (p = 0.4), based on a previous study by Shahvaran et al. [9]. Accordingly, q was taken as 0.6, with a 95% confidence level and a margin of error of ±10% (d = 0.1). Substituting these values:

$n = \frac{4 \times 0.4 \times 0.6}{(0.1)^2} = {96}$

Based on these parameters, the minimum required sample size was calculated to be approximately 96 participants. To enhance the robustness of the study and account for potential exclusions, a total of 103 patients with liver cirrhosis were ultimately enrolled.

Inclusion and exclusion criteria

Individuals aged 18 years and above diagnosed with decompensated liver cirrhosis, irrespective of etiology, were included in the study. Patients younger than 18 years or older than 70 years, as well as pregnant women, were excluded.

To minimize confounding cardiovascular factors, patients with known cardiomyopathy, heart failure, coronary artery disease, valvular heart disease, or documented arrhythmias were excluded based on clinical history, prior medical records, baseline electrocardiography (ECG), and echocardiographic evaluation. Patients with chronic anemia, systemic hypertension, other high-output cardiac states, chronic kidney disease, chronic respiratory disorders, or endocrine disorders that could independently affect cardiovascular function were also excluded from participation.

Ethical considerations

The Institutional Ethics Committee of SCB Medical College and Hospital, Cuttack, granted ethical approval for the study.

Data collection procedure

After receiving informed consent, 103 patients with hepatic cirrhosis were included. A thorough clinical evaluation was conducted, including a physical examination, medical history, and demographic information. Complete blood counts, liver and renal function tests, blood glucose levels, serum electrolytes, protein, albumin, and coagulation profiles were among the laboratory tests performed. Chest X-ray, urine analysis, abdominal ultrasonography, ascitic fluid analysis with serum-ascites albumin gradient computation, and upper gastrointestinal endoscopy were among the further studies. Diagnosis of cirrhosis was based on a combination of clinical findings, biochemical parameters, ultrasonographic evidence of chronic liver disease and portal hypertension, and supportive endoscopic findings where available. Consecutive eligible patients admitted to the departments of general medicine, hepatology, and cardiology during the study period were screened and enrolled after obtaining informed consent.

Assessment of cirrhosis severity and cardiovascular function

The severity of cirrhosis was assessed using the CTP scoring system [10,11], which incorporates clinical and biochemical parameters including ascites, hepatic encephalopathy, serum bilirubin, serum albumin, and prothrombin time. Patients were categorized into class A (5-6 points), class B (7-9 points), and class C (10-15 points).

Complete echocardiographic records suitable for detailed analysis of systolic and diastolic function were available for 40 patients. Cardiovascular assessment was performed using 12-lead ECG and transthoracic echocardiography (TTE). The QTc interval was calculated using Bazett’s formula:

$QT_c = \frac{QT}{\sqrt{RR}}$.

Cardiovascular assessment was performed using a 12-lead ECG and TTE. QTc values >440 ms were considered prolonged [12]. Echocardiographic evaluation included assessment of structural and functional parameters, including the E/A ratio, E/e′ ratio, end-diastolic volume, end-systolic volume, left ventricular ejection fraction (LVEF), tricuspid regurgitation velocity, and tricuspid annular plane systolic excursion. Systolic function was evaluated using LVEF calculated by Simpson’s method, while diastolic dysfunction was assessed using Doppler echocardiographic parameters in accordance with American Society of Echocardiography/European Association of Cardiovascular Imaging 2016 recommendations, including E/A ratio, E/e′ ratio, and supportive echocardiographic findings where available [13]. The study evaluated cardiovascular abnormalities associated with cirrhosis; however, complete fulfillment of the 2019 cirrhotic cardiomyopathy diagnostic criteria could not be uniformly assessed across all patients because advanced echocardiographic parameters were unavailable in all cases.

Statistical analysis

Data were analyzed using Microsoft Excel 2019 (Microsoft Corporation, Redmond, WA, USA) and the Statistical Package for the Social Sciences version 25.0 (IBM Corp., Armonk, NY). Categorical variables were expressed as frequencies and percentages, while continuous variables were presented as mean ± standard deviation.

Both descriptive and inferential statistical analyses were performed. The chi-square test was used to assess associations between categorical variables, and Pearson’s correlation coefficient was applied to evaluate relationships between continuous variables. A p value of <0.05 was considered statistically significant.

## Results

Demographic characteristics

The study population's average age was 44.22 ± 10.97 years. Most of the patients were between the ages of 40 and 60. There were 66 men (64%) and 37 women (36%), indicating a masculine preponderance. The age-wise distribution of the study participants is shown in Table 1.

**Table 1:** Age distribution of study participants

Age group (years)	Value, n (%)
<30	8 (7.8)
30-40	22 (21.4)
40-50	32 (31.1)
50-60	28 (27.2)
>60	13 (12.6)
Total	103 (100)

Gender distribution

There was a clear male predominance, with 66 men (64%) and 37 women (36%), as detailed in Table 2.

**Table 2:** Gender distribution

Gender	Value, n (%)
Male	66 (64)
Female	37 (36)
Total	103 (100)

Etiology of cirrhosis

Alcohol-related liver disease was the most common etiology, accounting for 72 patients (69.9%), followed by viral hepatitis in 16 patients (15.5%), nonalcoholic fatty liver disease in nine patients (8.7%), and other causes in six patients (5.8%), as shown in Table 3.

**Table 3:** Etiology of cirrhosis

Etiology	Value, n (%)
Alcoholic liver disease	72 (69.9)
Viral hepatitis	16 (15.5)
Nonalcoholic fatty liver disease	9 (8.7)
Others	6 (5.8)
Total	103 (100)

Severity of cirrhosis

Based on the CTP classification, the majority of patients were in advanced stages of disease: 46 patients (44.7%) in Class C, 43 (41.7%) in Class B, and 14 (13.6%) in Class A. The distribution according to the CTP class is presented in Table 4.

**Table 4:** Distribution according to Child-Turcotte-Pugh class

Child-Turcotte-Pugh class	Value, n (%)
A	14 (13.6)
B	43 (41.7)
C	46 (44.7)
Total	103 (100)

Systolic dysfunction

Systolic function assessment using LVEF was available for 40 out of 103 patients, as complete echocardiographic records suitable for detailed systolic function analysis were available for these patients. Systolic function assessment using LVEF was available for 40 out of 103 patients, as echocardiographic evaluation had been performed only in these patients during hospitalization/clinical workup, and complete echocardiographic records were retrievable for analysis. Among the assessed patients, the majority had preserved systolic function, with 35 patients (87.5%) classified as Heart Failure with preserved Ejection Fraction, while four patients (10%) had mildly reduced ejection fraction (Heart Failure with mildly reduced Ejection Fraction) and one patient (2.5%) had reduced ejection fraction (Heart Failure with reduced Ejection Fraction). The classification of systolic function based on LVEF is summarized in Table 5.

**Table 5:** Classification of systolic function based on LVEF HFpEF: Heart Failure with preserved Ejection Fraction; HFmrEF: Heart Failure with mildly reduced Ejection Fraction; HFrEF: Heart Failure with reduced Ejection Fraction; LVEF: left ventricular ejection fraction

Heart failure classification	Value, n (%)
HFpEF (≥50%)	35 (87.5)
HFmrEF (41%-49%)	4 (10)
HFrEF (≤40%)	1 (2.5)
Total	40 (100)

Diastolic dysfunction

Diastolic dysfunction was assessed in 40 patients with adequate echocardiographic data. Among these patients, diastolic dysfunction was observed in all 40 patients, to varying extents. The prevalence increased with worsening severity of cirrhosis. Among patients with diastolic dysfunction, six patients (15%) belonged to Child-Pugh Class A, 20 patients (50%) to Class B, and 14 patients (35%) to Class C, as shown in Table 6. Detailed grading of diastolic dysfunction and assessment of left ventricular filling pressures could not be uniformly evaluated because of incomplete echocardiographic data.

**Table 6:** Diastolic dysfunction according to Child-Pugh class

Child-Pugh class	Diastolic dysfunction present, n (%)
Class A	6 (15)
Class B	20 (50)
Class C	14 (35)
Total	40 (100)

A statistically significant association was observed between the severity of cirrhosis and the prevalence of diastolic dysfunction (p < 0.05).

Pulmonary hypertension

Pulmonary hypertension based on echocardiographic parameters was observed in four out of 40 patients (10%) who underwent echocardiographic evaluation. The diagnosis was based on echocardiographic findings suggestive of elevated pulmonary artery pressure. However, differentiation between pulmonary arterial hypertension and pulmonary venous hypertension secondary to left ventricular systolic or diastolic dysfunction could not be definitively established, as right heart catheterization was not performed.

Correlation between cirrhosis severity and cardiac dysfunction

Patients with severe cirrhosis were more likely to have cardiovascular problems. Patients with Child-Pugh class C cirrhosis had considerably more diastolic dysfunction than those in class A or B, suggesting a positive relationship between the severity of liver disease and cardiac dysfunction (Table 7).

**Table 7:** Pulmonary hypertension among study participants

Pulmonary hypertension	Value, n (%)
Present	4 (3.9)
Absent	99 (96.1)
Total	103 (100)

## Discussion

Liver cirrhosis is associated with significant systemic hemodynamic alterations that affect multiple organ systems. Cirrhotic cardiomyopathy is characterized by electrophysiological abnormalities, impaired diastolic relaxation, and a blunted cardiac contractile response despite the absence of primary cardiac disease. These changes are thought to result from chronic systemic vasodilation, neurohormonal activation, and progressive myocardial structural remodeling associated with advanced liver disease [14].

In the present study, most patients were middle-aged men, with alcohol-related liver disease being the predominant etiology. This demographic pattern is consistent with previous studies evaluating cardiovascular manifestations in cirrhosis, where alcohol-related cirrhosis commonly affects males in the productive age group [15].

The clinical profile of the study population was typical of decompensated cirrhosis, with ascites, jaundice, splenomegaly, and esophageal varices being frequently observed. Similar findings have been reported in earlier studies and reflect the systemic and portal hemodynamic consequences of advanced liver disease [16].

Electrocardiographic abnormalities, particularly QT interval prolongation, have been widely described in cirrhotic cardiomyopathy. Prolonged QT interval has been linked to autonomic dysfunction, altered ion channel activity, and increased sympathetic stimulation, potentially increasing the risk of arrhythmias in advanced cirrhosis [12].

Diastolic dysfunction was the most common cardiac abnormality identified in the present study and was more prevalent in patients with advanced CTP class. Similar observations have been reported previously, supporting the association between worsening liver dysfunction and impaired cardiac relaxation [17]. Structural myocardial changes such as fibrosis, hypertrophy, and reduced ventricular compliance are believed to contribute to abnormal ventricular filling patterns detectable on Doppler echocardiography [18]. Diastolic dysfunction is often considered the earliest manifestation of cirrhotic cardiomyopathy and may precede overt systolic impairment.

In contrast, systolic dysfunction was relatively uncommon, with most patients demonstrating preserved LVEF. This finding is consistent with previous literature suggesting that systolic dysfunction in cirrhosis may remain subclinical and become apparent only during physiological stress, including sepsis, gastrointestinal bleeding, surgery, or liver transplantation [19].

Pulmonary hypertension was identified in a small proportion of patients undergoing echocardiographic assessment. Portopulmonary hypertension is a recognized complication of portal hypertension and has important prognostic implications because it may increase perioperative risk and overall mortality [20]. Although the prevalence observed in the present study was low, similar findings have been reported in earlier echocardiographic studies of cirrhotic patients.

Overall, the findings of the present study demonstrate a significant association between increasing severity of cirrhosis and cardiovascular dysfunction, particularly diastolic dysfunction. Recognition of these cardiac abnormalities is clinically important because they may influence disease prognosis, therapeutic decisions, and pre-transplant evaluation in patients with advanced liver disease [20,21].

Limitations

The present study has several limitations. Because this is a single-center cross-sectional study, the findings may not be generalizable, and causal relationships cannot be established. The predominance of alcohol-related cirrhosis may limit extrapolation to other etiologies of liver disease. Complete echocardiographic records were available for only 40 patients, limiting comprehensive assessment of systolic and diastolic parameters in the entire study population. Severity grading of diastolic dysfunction and uniform application of contemporary cirrhotic cardiomyopathy diagnostic criteria could not be performed because advanced echocardiographic parameters were unavailable in some patients. Pulmonary hypertension assessment was based on echocardiographic findings alone, without right heart catheterization for definitive confirmation. Additionally, multivariate analysis was not performed because of the limited sample size and incomplete availability of certain variables. Therefore, the findings should be interpreted as exploratory observational data requiring further validation in larger prospective studies.

## Conclusions

The current study shows that one important but frequently overlooked consequence of liver cirrhosis is cardiovascular impairment. According to the CTP classification, diastolic dysfunction was the most often seen cardiac abnormality, and its incidence rose as liver disease severity worsened. The existence of electrophysiological anomalies, such as QT interval prolongation, suggests subclinical cardiac involvement in cirrhotic patients, even if systolic dysfunction was less commonly found. These results emphasize the significance of thorough cardiovascular assessment in cirrhosis patients, especially those with advanced illness. Early detection of heart dysfunction with noninvasive techniques like echocardiography and ECG may enable prompt intervention and enhance patient care in general. Further large-scale prospective studies are needed to better understand the clinical implications of cirrhotic cardiomyopathy and its impact on outcomes in patients with chronic liver disease.
