# Correction: Study of Cardiovascular Dysfunction in Cirrhosis and Its Correlation With Severity at a Tertiary Care Hospital

**DOI:** 10.7759/cureus.c443

**Published:** 2026-05-29

**Authors:** Bijaya K Behera, Romanchit Upadhyaya, Manasi Das, Sanatan Behera

**Affiliations:** 1 Medicine, Srirama Chandra Bhanja (SCB) Medical College and Hospital, Cuttack, IND; 2 Internal Medicine, Srirama Chandra Bhanja (SCB) Medical College and Hospital, Cuttack, IND; 3 Department of Neurology, IMS and SUM Hospital, Campus II, Cuttack, IND; 4 Gastroenterology, Srirama Chandra Bhanja (SCB) Medical College and Hospital, Cuttack, IND

This article has been corrected to change Dr. Manasi Das’s affiliation to: Department of Neurology, IMS and SUM Hospital, Campus II, Cuttack, India.

